# Perceived Stress Among Students and Their Perceptions of Mindfulness and Therapeutic Yoga Practice in Bhutan: A Comparative Cross‐Sectional Study

**DOI:** 10.1002/puh2.70348

**Published:** 2026-08-14

**Authors:** Phuntsho Wangdi, Choki Wangmo, Sonam Wangzin Rabjay, Sonam Lhundrup, Ugyen Chophel, Kinley Wangchuk, Karma Galey, Jamyang Yeshi Dorji, Thinley Dorji

**Affiliations:** ^1^ Traditional Medicine Unit Panbang Hospital Zhemgang Bhutan; ^2^ Faculty of Traditional Medicine Khesar Gyalpo University of Medical Sciences of Bhutan Thimphu Bhutan; ^3^ Department of General Practice Eastern Regional Referral Hospital Mongar Bhutan; ^4^ National Centre for Traditional Medicine Services Ministry of Health Thimphu Bhutan; ^5^ Department of General Practice Wangdue Hospital Wangdue Phodrang Bhutan; ^6^ National Traditional Medicine Hospital, Ministry of Health Thimphu Bhutan; ^7^ Department of Health Services Ministry of Health Thimphu Bhutan; ^8^ Department of Internal Medicine Central Regional Referral Hospital Gelephu Bhutan; ^9^ Faculty of Postgraduate Medicine Khesar Gyalpo University of Medical Sciences of Bhutan Thimphu Bhutan

**Keywords:** adolescent health, coping skills, mindfulness‐based cognitive therapy, stress, students

## Abstract

**Introduction:**

Student stress is an important public health concern. Mindfulness and Therapeutic Yoga Practice (*Sorig Zhiney and Luejong*) has been introduced in selected Bhutanese schools to promote student well‐being, but evidence regarding students’ perceived stress and experiences of these practices remains limited.

**Methods:**

This comparative cross‐sectional study included 1035 students in Classes IX–XII from four selected secondary schools in Punakha and Pemagatshel, Bhutan, 2025. Schools were purposively selected according to program implementation, and students were randomly selected from eligible student lists. Stress levels were measured using the Perceived Stress Scale (PSS), and factors associated with high perceived stress were identified using logistic regression.

**Results:**

Data from 490 students in schools implementing the program and 545 in schools without the program were analyzed. High perceived stress was reported by 28 (5.87%) students and 33 (6.25%) students, respectively (*p* = 0.946), mean PSS scores were similar (19.15 ± 4.87 vs. 19.18 ± 4.76; *p* = 0.910). Program availability was not associated with high perceived stress (adjusted odds ratio [OR] 0.91, 95% confidence interval [CI] 0.39–2.10; *p* = 0.823). Female sex, Class XII, very frequent disconnection from family, and poor appetite were associated with higher odds of high perceived stress. Among students in program schools, 392 (83.76%) practiced a few times weekly, 333 (68.38%) reported perceived benefits, and 435 (89.51%) recommended the practices to others.

**Conclusion:**

Students in schools with and without the program had similar levels of perceived stress. Students in program schools reported generally favorable perceptions and experiences.

## Graphical abstract

Students in schools with and without the Mindfulness and Therapeutic Yoga Practice program in Bhutan had similar levels of perceived stress. Students in program schools reported favorable perceptions and experiences of the practices, supporting the need for further longitudinal evaluation.

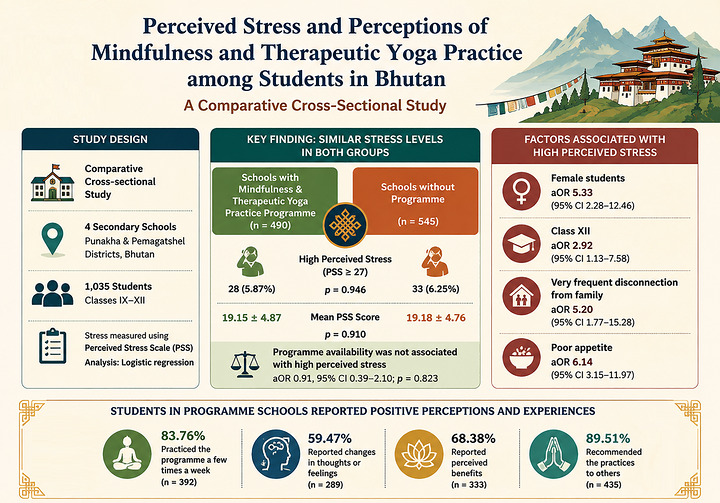

## 1 Introduction

Stress, as defined by the World Health Organization, is a state of worry or mental tension caused by a difficult situation [1]. The World Health Organization (2024) estimates that over 970 million people experience mental health conditions, with children and adolescents forming a substantial proportion of this population [2]. Nearly one in five adolescents experiences a mental health condition [2], and academic stress is a primary factor contributing to this burden [3, 4, 5, 6]. In today's highly competitive and fast‐paced world, students are increasingly exposed to a wide range of stressors from a young age. These include academic pressures, high parental expectations, peer competition, performance in standardized examinations, social comparison, and, in many cases, exposure to digital and social media [3, 4, 5, 6, 7, 8, 9, 10].

These challenges often result in detrimental outcomes such as poor academic performance and increased vulnerability to mental health conditions like anxiety and depression [3, 4, 5, 6, 7, 8, 9, 10]. In more severe cases, unmanaged stress can lead to behavioral issues, substance abuse, and even suicidal ideation among school‐aged children [5, 7, 11] and may also exhibit changes in appetite and quality of sleep [12, 13, 14]. Evidence from China indicates that the prevalence of psychological stress among secondary school adolescents ranges approximately from 12.9% to 26.6% [6]. Meanwhile, low‐ and middle‐income countries, despite having fewer structured mental health support systems, are witnessing a similar rise in adolescent stress levels [15].

In Bhutan, although the national development philosophy of Gross National Happiness (GNH) emphasizes holistic well‐being, student stress is becoming an increasingly visible concern. A study conducted among 638 secondary school students in Phuntshothang School, Punakha district, found that most students experienced moderate levels of stress. Common symptoms included insomnia, fatigue, anxiety, irritability, and a lack of motivation [7]. These manifestations highlight the need for early recognition of stress‐related signs and symptoms among students.

Existing literature on stress management among adolescents focuses predominantly on Western psychotherapeutic and pharmacological interventions, such as cognitive behavioral therapy, mindfulness‐based stress reduction activities, and medications [16]. Although evidence supports several of these approaches for managing stress, their accessibility, acceptability, and cultural relevance within Bhutanese school settings remain limited. This has prompted interest in exploring culturally grounded and locally acceptable approaches to student mental well‐being. In response, Bhutan has introduced Mindfulness and Therapeutic Yoga Practice (*Sorig Zhiney and Luejong*) in 2017, derived from Bhutanese Traditional Medicine (*Sowa Rigpa*), in selected schools [17]. Thise practice consists of structured mindfulness and physical exercises aimed at promoting mental calmness, emotional balance, and physical health and are aligned with the principles of GNH.

Despite the presence of such initiatives, empirical evidence on stress levels and the factors associated with high stress among secondary school students in Bhutan remains limited. Moreover, there is insufficient data comparing stress experiences across different school settings. This study, therefore, assessed the perceived stress, factors associated with high stress levels among students in selected secondary schools in Bhutan and compared perceived stress between students in schools with and without an established Mindfulness and Therapeutic Yoga Practice program. The study also assessed students’ perceptions and experiences of the practice.

## 2 Methods

### 2.1 Study Design

This comparative cross‐sectional study included students from four purposively selected schools across two districts in Bhutan. Two schools had an established Mindfulness and Therapeutic Yoga Practice program (*Sorig Zhiney and Luejong*), whereas two schools did not have the program.

### 2.2 Study Setting

#### 2.2.1 General Setting

In Bhutan, education is provided free of cost till Class XII by the Royal Government of Bhutan. As of 2024, the country had 566 schools in operation, including 540 government‐run and 26 privately managed, and served a total of 156,272 students [18]. Bhutan's education system consists of three levels: pre‐primary, secondary, and tertiary education. The pre‐primary level covers classes up to Class VI, the secondary level includes Classes VII–XII, and the tertiary level encompasses undergraduate and higher studies. Children enter pre‐primary at the age of five and progress through annual internal assessments until Class III. From Class IV onward, students are evaluated through yearly examinations, which determine their promotion to the next grade.

#### 2.2.2 Specific Setting

Mindfulness and Therapeutic Yoga Practice (*Sorig Zhiney and Luejong*) is a tradition derived from *Sowa Rigpa*, or Bhutanese Traditional Medicine. It includes 32 exercises under three categories: mental health, healthy living, and physical ailments. These exercises aim to enhance the functioning of the sensory and internal organs, balance internal energy, open energy channels, and calm the mind [17].

Introduced by the Ministry of Health as part of Bhutan's national wellness and spiritual health initiative, Mindfulness and Therapeutic Yoga Practice has been promoted in schools and institutions to improve the mental, physical, and spiritual well‐being of students and educators [17]. From 2017 to 2021, the Department of Traditional Medicine Services, Ministry of Health, had trained 421 individuals that include diverse profession, namely, traditional medicine professionals, personnel from the Royal Bhutan Police, persons with disabilities, civil servants, teachers, district health officers, and district education officers. Since its introduction in schools from 2022 in collaboration with the PEMA Secretariat and Ministry of Education, Mindfulness and Therapeutic Yoga Practice has gained popularity among students and teachers, who report perceived benefits.

### 2.3 Study Site

**FIGURE 1:**
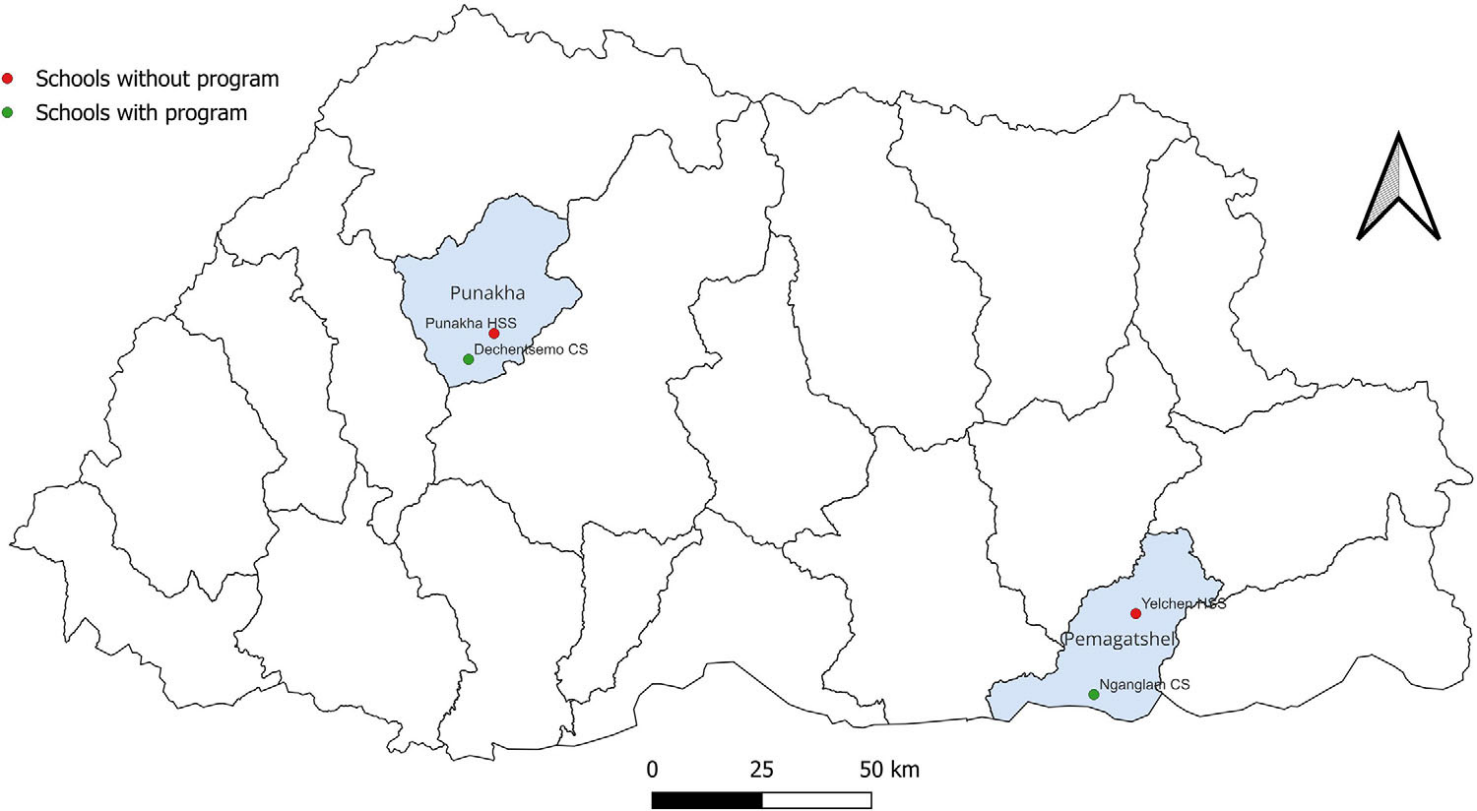
Study sites included for the assessment of perceived stress among students in selected schools in Punakha and Pemagatshel districts, Bhutan, 2025.

This study was carried out in selected schools located in two districts, Punakha and Pemagatshel districts. In each district, schools were purposively selected to include one school that has implemented the Mindfulness and Therapeutic Yoga Practice program and one school that has not. The selection was based on logistical feasibility and the willingness of school authorities to participate in the study (Figure 1).

### 2.4 Study Population, Sampling, and Sample Size

The sample selection was carried out using a multistage sampling approach. At the first stage, two districts were purposively selected: Punakha and Pemagatshel. From Punakha district, two schools were purposively selected: Dechentsemo Central School, which has implemented the Mindfulness and Therapeutic Yoga Practice program, and Punakha Central School, which has not. From Pemagatshel district, Nganglam Central School was selected, which has implemented the program, and Yelchen Central School, which has not.

The sample size was calculated using OpenEpi version 3.01. The calculation was based on proportions, assuming that 50% of students practicing the mindfulness and therapeutic yoga practices would report a reduction in stress levels. A margin of error of 5% and a design effect of 2 were applied to account for anticipated correlation among students within the schools, yielding a required sample size of 472 students per group. After adjusting for an anticipated nonresponse or dropout rate of 10%, the final estimated sample size was 524 students per group (Table S1a,b). The total sample size was 1048 students.

From each school, the list of students in each class level was combined to generate the total list of students with designated serial numbers. A random sequence generator was then used to select the required number of students. In cases where the desired student was absent on the day of the interview, the next student on the random sequence list was selected. Students who gave assent and had parental/guardian consent (if under 18 years) and students who were present at school during the data collection were included in the study.

### 2.5 Study Period

The study was conducted over 2 months from October to November, 2025.

### 2.6 Data Variables and Study Tool

Data were gathered using Google Forms in schools where information technology laboratories were available; in schools without access to electronic facilities, paper‐based questionnaires were used..

A structured questionnaire was used as the primary study tool. It comprised three parts. Part A: sociodemographic characteristics of the participants (age, sex, class, their current living arrangement, number of parents/family members visiting, feeling disconnected from family/parents, pocket money, use of mobile phones, stress, sleep, appetite, hospital visits, and the type of treatment received). Part B: Perceived stress, assessed using the internationally validated Perceived Stress Scale‐10 (PSS‐10) [19]. Part C: Frequency of Mindfulness and Therapeutic Yoga Practice undertaken in the school, changes in thoughts after practicing it, whether it was helpful.

Perceived Stress Scale (PSS‐10), a widely recognized, self‐administered psychological tool designed to measure the perception of stress [19]. The PSS‐10 consists of 10 items divided into two sub‐scales: Perceived Helplessness (Items 1, 2, 3, 6, 9, and 10), which reflects an individual's sense of losing control over situations or emotions, and Lack of Self‐Efficacy (Items 4, 5, 7, and 8), which measures one's perceived inability to cope with challenges. The reliability of the PSS‐10 has been well established, with Cronbach's alpha exceeding 0.70 across 12 validation studies, and test–retest reliability also meeting the >0.70 criterion in four studies. Each item on the scale is scored from 0 (never) to 4 (very often), resulting in a total score ranging from 0 to 40, where higher scores indicate greater perceived stress. For interpretative purposes, scores are categorized as follows: 0–13 indicates low stress, 14–26 indicates moderate stress, and 27–40 indicates high perceived stress.

Content validation was performed for Parts A and C by a panel of nine experts, comprising two teachers, three general duty medical officers (GDMOs), one clinical nurse, one physiotherapist, and two traditional medicine physicians (*drungtshos*). During the first round, seven items did not meet the required threshold of ≥75% inter‐rater agreement. After careful discussion, three items were dropped, and the remaining four were revised on the basis of the feedback received from experts. In the second round of evaluation, all items met the threshold with individual item scores ≥75%. The Scale‐Content Validation Index was 94.5%.

### 2.7 Data Analysis and Statistics

Data were collected and entered into Google Forms. Data analysis was performed using STATA (version 18.0, StataCorp LP, USA, licensed by Khesar Gyalpo University of Medical Sciences of Bhutan). Categorical variables were summarized using frequency and percentage, and continuous variables were summarized using mean and standard deviation.

The PSS‐10 score was presented using mean and standard deviation. Differences in the mean PSS‐10 scores between schools with and without the Mindfulness and Therapeutic Yoga Practice program were assessed using an independent *t*‐test. Factors associated with “high perceived stress” on PSS‐10 were assessed using multivariable logistic regression. Those factors with *p* < 0.100 on unadjusted analyses were selected for adjusted analysis, and adjusted odds ratios (ORs) with 95% confidence intervals (CIs) are reported. Factors with *p* < 0.05 were considered significant. Missing observations were excluded from analyses involving the respective variables using complete‐case analysis. The number of missing observations for individual variables is reported in the tables.

As a sensitivity analysis, the multivariable logistic regression model was additionally fitted with standard errors clustered at the school level. As the study included only four schools, conventional cluster‐robust inference may be unreliable with such a small number of clusters. Therefore, wild cluster bootstrap inference using Webb weights was additionally performed to assess the robustness of the regression findings to school‐level clustering. The sensitivity analysis used the same covariates as the primary multivariable model.

## 3 Results

There were 1035 students included in the study (response rate 98.75%): 490 students in schools implementing the program and 545 students in schools without the program. The mean age was 17 (±1.51) years, range 14–23 years. The basic characteristics of the students, living arrangements, frequency of parental visits, use of mobile phone, patterns of appetite, and frequency of hospital visits are shown in Table 1.

**TABLE 1:** Sociodemographic characteristics and stress levels among students in selected schools in Punakha and Pemagatshel districts, Bhutan, 2025 (*n* = 1035).

		Schools with Mindfulness and Therapeutic Yoga Practice program		Schools without Mindfulness and Therapeutic Yoga Practice program		
Characteristics	Total	*n*	%	*n*	%	*p* value
Sex						
Male	478	227	47.49	251	52.51	0.930
Female	557	263	47.22	294	52.78	
Class						
Class IX	213	169	79.34	44	20.66	**<0.001**
Class X	278	143	51.44	135	48.56	
Class XI	204	69	33.82	135	66.18	
Class XII	340	109	32.06	231	67.94	
Living arrangements						
Boarding	662	312	47.13	350	52.87	0.610
Friends	1	—	—	1	100.00	
Parents	311	151	48.44	160	51.45	
Relatives	53	25	47.17	28	52.83	
Not specified	8	2	25.00	6	75.00	
Frequency of parents’ visit if boarding (*n* = 662)						
Never	46	15	32.61	31	67.39	0.031
Few times in a month	492	235	47.76	257	52.24	
Few times a year	78	28	35.90	50	64.10	
How often, students feel disconnected from parents^a^						
Very often	37	11	29.73	26	70.27	0.008
Sometimes	441	223	50.57	218	49.43	
Often	78	44	56.41	34	43.59	
Never	462	201	43.51	261	56.49	
Receiving pocket money from parents/family members^b^	949	439	46.26	510	52.89	0.046
Using a mobile phone distracts or increases their stress levels^c^						
Yes, often	141	44	31.21	97	68.79	**<0.001**
Sometimes	367	206	56.13	161	43.87	
Rarely	235	116	49.36	119	50.64	
Never	288	122	42.36	166	57.64	
Use of a mobile phone increases stress, distracts from studies and mindfulness activities^d^						
Yes, often	138	56	40.58	82	59.42	**<0.001**
Sometimes	398	213	53.52	185	46.48	
Rarely	234	119	50.85	115	49.15	
Never	260	99	38.08	161	61.92	
Sleeps well^e^	869	400	46.67	469	53.97	**0.014**
Waking up feeling tired or unrested even after a full night's sleep^f^						
Yes, often	183	83	45.36	100	54.64	0.185
Sometimes	474	226	47.68	248	52.32	
Rarely	231	100	43.29	131	56.71	
Never	139	76	54.68	63	45.32	
Appetite for meals^g^						0.209
Good appetite	790	376	47.59	414	52.41	
Not having a good appetite currently	173	75	43.35	98	56.65	
Do not want to answer	66	37	56.06	29	43.94	
Hospital visits with body pains and aches in the last three months (*n* = 286)^h^		138	48.25	148	51.75	0.742
Up to 4 visits	150	83	55.33	67	44.67	0.105
5–8 visits	16	12	75	4	25	
9–12 visits	3	3	100	—	—	
Treatment during hospital visits						
Counseling or psychological support	19	7	36.84	12	63.16	0.181
Stress management sessions	7	4	57.14	3	42.86	
Meditation and mindfulness	21	12	52.17	11	47.83	
Medication	203	104	51.23	99	48.77	
No intervention	27	18	50	18	50	
Perceived Stress Scale score^i^						
High stress (27–40)	61	28	45.90	33	54.10	0.946
Moderate stress (14–26)	839	398	47.44	441	52.56	
Low stress (0–13)	105	51	48.57	54	51.43	

*Note:* bold values indicate significant *p*< 0.05.

^a^ Missing = 1.

^b^ Missing = 16.

^c^ Missing = 4.

^d^ Missing = 5.

^e^ Missing = 7.

^f^ Missing = 8.

^g^ Missing = 6.

^h^ Missing = 117.

^i^ PSS score; Min. = 0, Max. = 40.

### 3.1 Factors Associated With Levels of Stress

**FIGURE 2:**
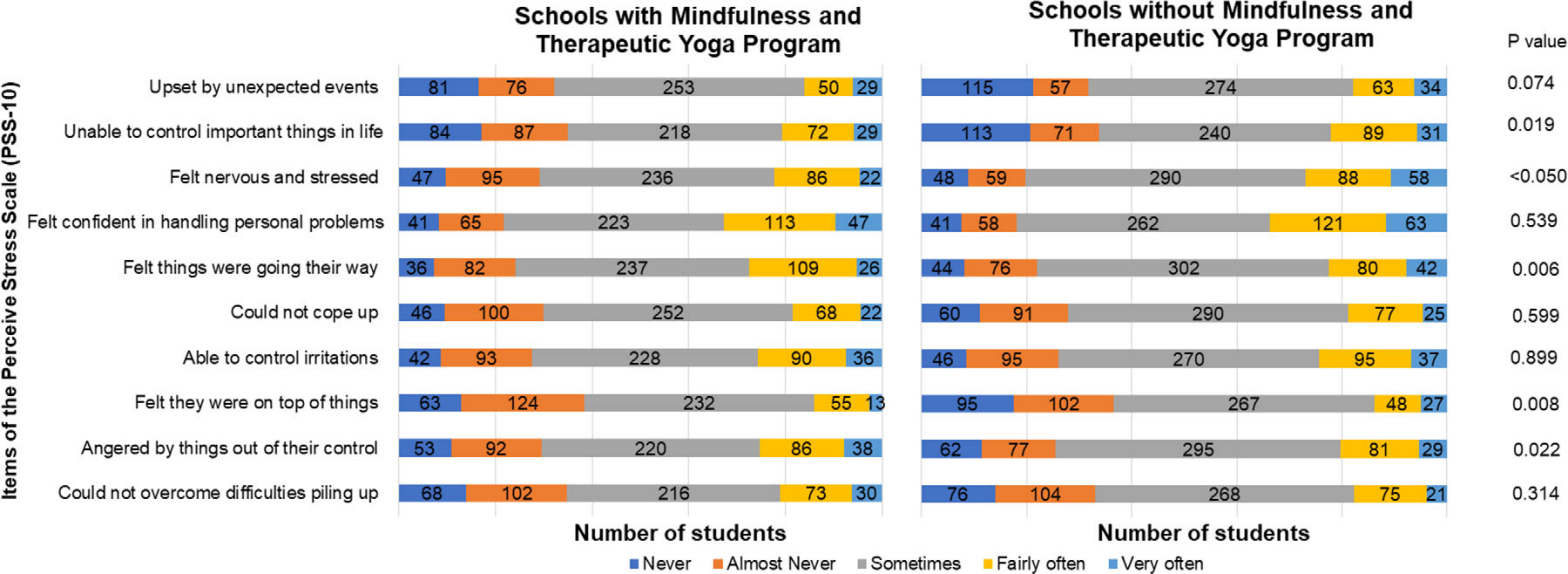
Item‐wise distribution responses to the 10‐item Perceived Stress Scale (PSS) among students in selected schools in Punakha and Pemagatshel districts, Bhutan, 2025.

In schools where the program was implemented, there were 28 (5.87%) students with high stress, 398 (83.44%) with moderate stress, and 51 (10.69%) with low stress. In schools where the program was not implemented, there were 33 (6.25%) students with high stress, 441 (83.52%) with moderate stress, and 54 (10.23%) with low stress, *p* = 0.946 (Table 1). Among students studying in the school where the program was implemented, the mean PSS score was 19.15 (±4.87), and in school where the program was not implemented, the mean was 19.18 (±4.76), *p* = 0.910. The details of the item‐wise assessment of stress are shown in Figure 2 (Table S2).

In schools with the program, 392 (83.76%) practiced it a few times a week, 289 (59.47%) reported changes in thought/feelings, 333 (68.38%) reported positive benefit, and 435 (89.51%) recommended the practice to others. The details of practices and the impact of the program are shown in Table 2.

**TABLE 2:** Mindfulness and Therapeutic Yoga Practice (*Sorig Zhiney and Luejong*) among students in selected schools in Punakha and Pemagatshel, Bhutan, 2025 (*n* = 490).

Mindfulness and Therapeutic Yoga Practice	*n*	%
Frequency of practice (*n* = 490)^a^		
Everyday	59	12.61
Few times a week	392	83.76
Few times a month	8	1.71
Few times a year	9	1.92
Any noticeable changes in thoughts/feelings after practicing^b^		
Yes	289	59.47
No	58	11.93
Don't know	139	28.60
If the practice had helped in any form^c^		
Yes	333	68.38
No	31	6.37
Don't know	123	25.26
Recommendation of Mindfulness and Therapeutic Yoga Practice to others^b^	435	89.51

^a^ Missing = 22.

^b^ Missing = 4.

^c^ Missing = 3.

The multivariable logistic regression model included 914 participants with complete data for all variables included in the model. Overall, female students (adjusted OR 5.33, 95% CI 2.28–12.46, *p* < 0.001), students in Class XII (adjusted OR 2.92, 94% CI 1.12–7.58, *p* = 0.027), feeling disconnected “very often” from family (adjusted OR 5.20, 95% CI 1.77–15.38, *p* = 0.003), and those not having a good appetite (adjusted OR 6.14, 95% CI 3.15–11.97, *p* < 0.001) had higher odds of high perceived stress. There were no differences in the level of perceived stress with the practice of Mindfulness and Therapeutic Yoga Prarogram. The details of factors associated with reported high levels of stress are presented in Table 3 (subgroup analyses are shown in Tables S3 and S4).

**TABLE 3:** Factors associated with high perceived stress among students in selected schools in Punakha and Pemagatshel districts, Bhutan, 2025.

		Unadjusted analysis			Adjusted analysis		
Characteristics^a^	Total	OR	95% CI	*p* value	Adjusted OR	95% CI	*p* value
Age (years)							
≥14 to <16	90	Ref.					
≥16 to <18	400	1.36	0.39–4.72	0.630			
≥18	545	2.29	0.69–7.57	0.174			
Sex							
Male	478	Ref.			Ref.		
Female	557	6.14	2.88–13.05	<0.001	5.33	2.28–12.46	**<0.001**
Class							
Class IX	213	Ref.			Ref.		
Class X	278	1.31	0.51–3.40	2	1.01	0.33–3.09	0.984
Class XI	204	0.59	0.17–2.03	0.400	0.33	0.06–1.70	0.183
Class XII	340	3.64	1.59–8.32	0.002	2.92	1.12–7.58	**0.027**
Living arrangement							
Parents	311	Ref.					
Boarding	662	0.73	0.42–1.27	0.267			
Friends	1	1	—	—			
Relatives	53	0.79	0.23–2.76	0.719			
Not specified	8	2.12	0.24–18.41	0.495			
Feeling disconnected from family							
Never	462	Ref.			Ref.		
Very often	37	5.66	2.31–13.88	<0.001	5.20	1.77–15.28	**0.003**
Often	78	3.42	1.58–7.42	0.002	2.58	0.95–7.02	0.063
Sometimes	441	1.07	0.58–1.99	0.826	0.98	0.47–2.04	0.963
Receives pocket money	949	0.79	0.31–2.07	0.644			
Distraction or increase in stress with phone use							
Never	288	Ref.					
Yes, often	141	1.87	0.86–4.06	0.111			
Sometimes	367	1.12	0.57–2.21	0.741			
Rarely	235	0.89	0.39–1.97	0.768			
Increase in stress with use of phone							
Never	260	Ref.					
Yes, often	138	1.66	0.77–3.60	0.197			
Sometimes	398	1.01	0.52–1.98	0.975			
Rarely	234	0.73	0.32–1.67	0.462			
Good sleep							
Yes	869	Ref.			Ref.		
No	159	2.41	1.34–4.36	0.003	1.15	0.51–2.55	0.738
Appetite							
Good appetite	790	Ref.			Ref.		
Not having good appetite	173	7.79	4.38–13.86	<0.001	6.14	3.15–11.97	**<0.001**
Mindfulness and Therapeutic Yoga Practice							
No	158	Ref.					
Yes	874	0.48	0.26–0.88	0.017	0.91	0.39–2.99	0.823

*Note:* bold values indicate significant *p*< 0.05.

^a^ Multivariable analysis was based on complete cases (*n* = 914). A sensitivity analysis accounting for school‐level clustering was performed using school‐clustered standard errors and wild cluster bootstrap inference with Webb weights because only four schools were included. The sensitivity analysis attenuated statistical significance for several associations; bootstrap *p* values were 0.112 for sex, 0.054 for appetite, 0.425 for class (joint test), 0.336 for family disconnection (joint test), and 0.605 for Mindfulness and Therapeutic Yoga Practice.

Abbreviations: CI, confidence interval; OR, odds ratio.

## 4 Discussion

This comparative cross‐sectional study assessed the perceived stress and the factors associated with high stress among students in four selected secondary schools in Bhutan, including schools with and without an established Mindfulness and Therapeutic Yoga Practice program. A high proportion of students reported moderate stress levels, a finding that was consistent with most of the studies [6, 7, 8]. There was no statistically significant difference in the mean scores of perceived stress between students in schools implementing the Mindfulness and Therapeutic Yoga Practice program and those in schools without the program, suggesting that the intervention may not have produced a large effect on overall stress levels at the school population level over the study period. Given the cross‐sectional design and the non‐random selection of schools, this finding should be interpreted as an observed difference in stress levels between the two groups rather than evidence regarding the effectiveness or lack of effectiveness of the program.

Despite the absence of a statistically significant difference in perceived stress between the two groups, students in the schools implementing the program reported positive subjective experiences. Most students reported practicing the exercises a few times per week, and over half perceived improvements in their thoughts, feelings, and emotional regulation following participation. Furthermore, the majority considered the program beneficial and expressed willingness to recommend it to peers, indicating high acceptability and engagement. These findings suggest that although measurable reductions in stress may be modest or not immediately evident, mindfulness‐based programs are feasible, acceptable, and perceived as helpful, supporting their continued use in school settings.

These findings align with broader evidence from larger trials and pilot testing on the impact of yoga and meditation on the levels of stress [20]. For instance, a randomized controlled trial in 2025 found that 8‐week structured mindfulness training significantly decreased academic stress among university students while also lowering academic burnout and improving psychological resilience [21]. These results highlight the potential impact of mindfulness practices to confer mental health benefits, particularly when practiced regularly. Systematic reviews and meta‐analyses further indicate moderate evidence for reductions in anxiety and depression, but only low or inconsistent evidence for stress reduction, with no clear superiority over other active interventions such as exercise or cognitive‐behavioral therapy [22]. Differences in study design, population, intervention characteristics, and outcome assessment should be considered when comparing these findings with the present study.

In this study, female students were found to have higher odds of experiencing high stress, a finding that is consistent with most existing literature suggesting that adolescent girls may be more vulnerable to stress due to biological, emotional, and sociocultural factors [6, 7, 8]. This highlights the importance of gender‐sensitive mental health interventions within school settings.

The study also revealed that Class XII had higher odds of high perceived stress, adjustment for class resulted in only a modest change in the program‐stress association, which aligns with global studies showing that academic pressures are a major source of stress among adolescents [6, 10]. In many Asian cultures, including Bhutan, academic performance is highly emphasized, and both parents and teachers place considerable expectations on students to excel for their future opportunities. In Bhutan, Class XII represents a critical turning point, as students’ career paths are largely determined by the results of the Bhutanese board examinations, which are also a key source of stress.

Another significant factor associated with high stress was students who reported feeling disconnected from their families very often. Evidence from China identified family‐related factors as significant contributors to high stress in adolescents [6]. Family conflict or a poor parent–child relationship weakens a child's sense of closeness and belonging within the family. This lack of connection negatively impacts mental health and increases psychological stress, highlighting the critical role of family support in maintaining adolescents’ psychological well‐being [6]. Poor appetite was also associated with high stress levels. Students who reported not having a good appetite were more likely to experience high stress. This highlights the significance of appetite as both a potential sign and consequence of stress. Changes in appetite may reflect physiological and psychological responses to stress. Previous studies have suggested a bidirectional relationship between stress and appetite, where stress can alter eating patterns and, conversely, irregular eating can contribute to higher stress levels [6, 9, 14].

Although school‐based mindfulness programs, such as Mindfulness and Therapeutic Yoga Practice, are valuable and well‐received, academic pressures, family connectedness, and lifestyle factors such as appetite all play important roles in stress perceptions. Such programs may serve as supportive tools, fostering positive mental health perceptions, enhancing coping strategies, and improving self‐awareness among students. However, the present study cannot determine whether the program itself reduces stress or improves mental well‐being. Longitudinal or controlled intervention studies are needed to assess the effectiveness of the program and to determine whether sustained participation is associated with changes in perceived stress over time. Future research should prioritize larger, well‐powered studies with longer intervention durations and explore the integration of multimodal approaches, combining mindfulness, cognitive‐behavioral therapy, and physical activity to optimize stress reduction and overall well‐being.

### 4.1 Limitations

This study has several limitations. First, the study was conducted in only four selected secondary schools out of all secondary schools in Bhutan. Although a design effect of 2 was incorporated into the sample‐size calculation to account for clustering, only four independent school‐level clusters were available for analysis. Consequently, school‐clustered statistical inference has limited precision and statistical power. The wild cluster bootstrap sensitivity analysis also demonstrated attenuation of statistical significance for several associations. Because the program exposure was determined at the school level, the association between program participation and perceived stress cannot be commented on reliably.

Second, the comparative cross‐sectional design does not establish temporal or causal relationships between program availability and perceived stress. Third, responses related to perceived benefits and recommendations of the program may be subject to response bias, as students might have felt inclined to provide favorable answers. Fourth, although the study compared schools implementing and not implementing program, variations in duration, frequency, and individual adherence to the program were not fully captured, which may have influenced the findings. Fifth, the outcome of stress was a self‐reported one that did not allow further exploration of the level of stress and factors associated with it. Therefore, further studies with qualitative exploration are recommended to evaluate the factors associated with mental stress and the potential therapeutic effects of this program on the reduction of stress levels in students.

## 5 Conclusions

The study found no statistically significant difference in perceived stress between students in schools with and without the Mindfulness and Therapeutic Yoga Practice program. Students in schools with the program nevertheless reported generally positive perceptions of the practices and a high willingness to recommend them to others. Female students, students in Class XII, students who frequently felt disconnected from their families, and students with poor appetite showed higher odds of high perceived stress. Longitudinal and controlled studies should evaluate whether participation in Mindfulness and Therapeutic Yoga Practice is associated with changes in perceived stress over time and determine its effectiveness.

## Author Contributions

**Phuntsho Wangdi**, **Choki Wangmo**, **Sonam Wangzin Rabjay**, **Sonam Lhundrup**, **Ugyen Chophel**, **Kinley Wangchuk**, **Karma Galey**, **Jamyang Yeshi Dorji**, and **Thinley Dorji** were involved in the conceptualization, methodology, investigation, resources, writing – original draft, and writing – review and editing. Choki Wangmo, Phuntsho Wangdi, and Sonam Lhundrup were involved in data curation and project administration. Choki Wangmo, Karma Galey, Ugyen Chophel, and Thinley Dorji were involved in validation, software, formal analysis, and visualization. Thinley Dorji was involved in supervision. Sonam Lhundrup was involved in making arrangements for resources. All authors reviewed and approved the manuscript.

## Funding

The authors have nothing to report.

## Ethics Statement

This study was conducted according to the principles of the Declaration of Helsinki. Ethics approval was obtained from the Research Ethics Board of Health, Ministry of Health, Bhutan (Ref. No./PO/RL/2025.32.NNW, dated 26 September 2025). Administrative approval was obtained from the Policy and Planning Division, Ministry of Health. Permission to conduct this study was obtained from the Ministry of Education and Skills Development, the District Education Officer, and the respective principals of the school.

## Consent

Informed written consent was taken from students aged ≥18 years. Informed written assent was taken from students aged <18 years along with informed consent from their legal guardians.

## Conflicts of Interest

The authors declare no conflicts of interest.

## Supporting information




**Supporting File 1**: puh270348‐sup‐0001‐SuppMat.docx

## Data Availability

Data set is available from the corresponding author upon reasonable request and after permission from the Ministry of Health, Royal Government of Bhutan.

## References

[1] World Health Organization , Stress (World Health Organization, 2026), https://www.who.int/news‐room/questions‐and‐answers/item/stress.

[2] World Health Organization , World Mental Health Report: Transforming Mental Health for All (World Health Organization, 2022).

[3] T. Steare , C. Gutiérrez Muñoz , A. Sullivan , and G. Lewis , “The Association Between Academic Pressure and Adolescent Mental Health Problems: A Systematic Review,” Journal of Affective Disorders 339 (2023): 302–317, 10.1016/j.jad.2023.07.028. 37437728

[4] T. Xu , F. Zuo , and K. Zheng , “Parental Educational Expectations, Academic Pressure, and Adolescent Mental Health: An Empirical Study Based on CEPS Survey Data,” International Journal of Mental Health Promotion 26 (2024): 93–103, 10.32604/ijmhp.2023.043226.

[5] M. C. Pascoe , S. E. Hetrick , and A. G. Parker , “The Impact of Stress on Students in Secondary School and Higher Education,” International Journal of Adolescence and Youth 25 (2020): 104–112, 10.1080/02673843.2019.1596823.

[6] L. Hao , A. F. Mat Ludin , M. Ahmad , X. Meng , and H. Zhong Lei , “The Prevalence and Its Associated Factors of Psychological Stress Among Middle School Students in China: Pooled Evidence From a Systematic Scoping Review,” Frontiers in Public Health 12 (2024): 1358210, 10.3389/fpubh.2024.1358210. 38694991 PMC11062323

[7] Y. Nidup , D. R. Chetri , S. Wangchuk , T. Jamtsho , and B. Bagawath , “Perceived Stress of High School Students,” Journal of Research in Social Sciences and Language 2 (2022): 95–107, 10.71514/jssal/2022.51.

[8] A. E. Bezie , G. Abere , G. T. Zewude , et al., “Prevalence of Stress and Associated Factors Among Students in Ethiopia: A Systematic Review and Meta‐Analysis,” Frontiers in Public Health 13 (2025): 1518851, 10.3389/fpubh.2025.1518851. 40034176 PMC11874314

[9] I. M. Shatwan and M. A. Alzharani , “Association Between Perceived Stress, Emotional Eating, and Adherence to Healthy Eating Patterns Among Saudi College Students: A Cross‐Sectional Study,” Journal of Health, Population, and Nutrition 43 (2024): 144, 10.1186/s41043-024-00637-w. 39252087 PMC11385838

[10] K. Wunsch , J. Fiedler , P. Bachert , and A. Woll , “The Tridirectional Relationship Among Physical Activity, Stress, and Academic Performance in University Students: A Systematic Review and Meta‐Analysis,” International Journal of Environmental Research and Public Health 18 (2021): 739, 10.3390/ijerph18020739. 33467118 PMC7830011

[11] T. Dema , J. P. Tripathy , S. Thinley , et al., “Suicidal Ideation and Attempt Among School Going Adolescents in Bhutan—A Secondary Analysis of a Global School‐Based Student Health Survey in Bhutan 2016,” BMC Public Health 19 (2019): 1605, 10.1186/s12889-019-7791-0. 31791280 PMC6889681

[12] P. J. Kennelly , K. M. Botham , O. McGuinness , V. W. Rodwell , and P. A. Weil , Harper's Illustrated Biochemistry, 32nd ed. (McGraw Hill, 2022).

[13] B. Thapa , S. Chaudhary , R. Karki , et al., “Sleep Quality Is Associated With Stress in Secondary School Students,” BMC Public Health 25 (2025): 1397, 10.1186/s12889-025-22652-0. 40229759 PMC11995653

[14] I. F. Muhaimin , D. I. Puspitasari , E. N. Widiyaningsih , and L. Hidayati , “Correlation Between Stress Levels and Eating Behavior in College Students: A Study at The Faculty of Health Sciences,” Jurnal Gizi Prima (Prime Nutrition Journal) 8 (2023): 107–115, 10.32807/jgp.v8i2.401.

[15] F. Mascayano , J. E. Armijo , and L. H. Yang , “Addressing Stigma Relating to Mental Illness in Low‐ and Middle‐Income Countries,” Front Psychiatry 6 (2015): 38, 10.3389/fpsyt.2015.00038. 25814959 PMC4355984

[16] G. González‐Valero , F. Zurita‐Ortega , J. L. Ubago‐Jiménez , and P. Puertas‐Molero , “Use of Meditation and Cognitive Behavioral Therapies for the Treatment of Stress, Depression and Anxiety in Students. A Systematic Review and Meta‐Analysis,” International Journal of Environmental Research and Public Health 16 (2019): 4394, 10.3390/ijerph16224394. 31717682 PMC6888319

[17] The PEMA , Sorig Zhiney and Luejong Training, Eastern Region (PEMA, 2023), https://thepema.gov.bt/sorig‐zhiney‐and‐luejong‐training‐eastern‐region/.

[18] Ministry of Education and Skills Development , Annual Education Statistics (Ministry of Education and Skills Development, 2024).

[19] S. Cohen , Perceived Stress in a Probability Sample of the United States. The Social Psychology of Health (Sage Publications, Inc, 1988).

[20] V. Lemay , J. Hoolahan , and A. Buchanan , “Impact of a Yoga and Meditation Intervention on Students' Stress and Anxiety Levels,” American Journal of Pharmaceutical Education 83 (2019): 7001, 10.5688/ajpe7001. 31333265 PMC6630857

[21] S. Chen and X. Qi , “A Randomized Controlled Trial of Mindfulness: Effects on Academic Stress, Academic Burnout, and Psychological Resilience in University Students,” Frontiers in Psychology 16 (2025): 1722669, 10.3389/fpsyg.2025.1722669. 41394033 PMC12696859

[22] M. Goyal , S. Singh , E. M. S. Sibinga , et al., “Meditation Programs for Psychological Stress and Well‐Being: A Systematic Review and Meta‐Analysis,” JAMA Internal Medicine 174 (2014): 357–368, 10.1001/jamainternmed.2013.13018. 24395196 PMC4142584

