# Supplementary material for: Perceived Stress Among Students and Their Perceptions of Mindfulness and Therapeutic Yoga Practice in Bhutan: A Comparative Cross‐Sectional Study
Source: Public Health Chall. 2026 Aug 14;5(3):e70348. doi: 10.1002/puh2.70348 (PMC13475629; doi:10.1002/puh2.70348)
Supplement: Supplementary file 1 [file PUH2-5-e70348-s001.docx]

**Supplementary Table 1a. Sampling of students from schools that practice Mindfulness and Therapeutic Yoga Practice (*Sorig Zhiney* and *Luejong)*, 2025**

| **Class** | **Dechentsemo CS** | | | **Nganglam CS** | | | **Total sample (n1 + n2)** |
| --- | --- | --- | --- | --- | --- | --- | --- |
|  | **No of students** | **Proportion (%)** | **Sample (n1)** | **No of students** | **Proportion (%)** | **Sample (n2)** |  |
| IX | 99 | 40 | **85** | 123 | 35 | **107** | **192** |
| X | 62 | 25 | **53** | 69 | 19 | **58** | **111** |
| XI | 45 | 18 | **38** | 109 | 30 | **92** | **130** |
| XII | 44 | 17 | **37** | 59 | 16 | **50** | **85** |
| Total | 250 | 100 | **213** | 360 | 100 | **307** | **520** |

**Supplementary Table 1b. Sampling of students that do not practice Mindfulness and Therapeutic Yoga Practice, (*Sorig Zhiney* and *Luejong), 2025***

| **Class** | **Punakha Central School** | | | **Yelchen Central School** | | | **Total Sample**  **(n1 + n2)** |
| --- | --- | --- | --- | --- | --- | --- | --- |
|  | **No. of students** | **Proportion**  **%** | **Sample (n1)** | **No. of students** | **Proportion**  **%** | **Sample (n2)** |  |
| IX | 65 | 16 | **49** | 64 | 23 | **50** | **99** |
| X | 67 | 17 | **52** | 46 | 16 | **35** | **87** |
| XI | 147 | 38 | **115** | 81 | 29 | **62** | **177** |
| XII | 115 | 29 | **88** | 90 | 32 | **69** | **157** |
| Total | 394 | 100 | **304** | 281 | 100 | **216** | **520** |

Supplementary Table 2. Item-wise distribution of responses to the 10-item Perceived Stress Scale (PSS) among students in schools where the program was implemented and not implemented

| Perceived Stress Scale Questions | Students who practice | | | | | | | | | | Students who do not practice | | | | | | | | | |  |
| --- | --- | --- | --- | --- | --- | --- | --- | --- | --- | --- | --- | --- | --- | --- | --- | --- | --- | --- | --- | --- | --- |
|  | Never | | Almost Never | | Sometimes | | Fairly often | | Very often | | Never | | Almost Never | | Almost Never | | Fairly often | | Very often | |  |
|  | n | % | n | % | n | % | n | % | n | % | n | % | n | % | n | % | n | % | n | % | P value |
| Upset by unexpected events | 81 | 41.33 | 76 | 57.14 | 253 | 48.01 | 50 | 44.25 | 29 | 46.03 | 115 | 58.67 | 57 | 42.86 | 274 | 51.99 | 63 | 55.65 | 34 | 53.97 | 0.074 |
| Unable to control important things in life | 84 | 42.64 | 87 | 55.06 | 218 | 47.6 | 72 | 44.72 | 29 | 48.33 | 113 | 57.36 | 71 | 44.94 | 240 | 52.4 | 89 | 55.28 | 31 | 51.67 | 0.199 |
| Felt nervous and stressed | 47 | 49.47 | 95 | 61.69 | 236 | 44.87 | 86 | 49.43 | 22 | 27.5 | 48 | 50.53 | 59 | 38.31 | 290 | 55.31 | 88 | 50.57 | 58 | 72.5 | 0 |
| Felt confident in handling personal problems | 41 | 50 | 65 | 52.85 | 223 | 45.98 | 113 | 48.29 | 47 | 42.73 | 41 | 50 | 58 | 47.15 | 262 | 54.02 | 121 | 51.71 | 63 | 57.27 | 0.539 |
| Felt things were going their way | 36 | 45 | 82 | 51.9 | 237 | 43.97 | 109 | 57.67 | 26 | 38.24 | 44 | 55 | 76 | 48.1 | 302 | 56.03 | 80 | 42.33 | 42 | 61.76 | 0.006 |
| Could not cope up | 46 | 43.4 | 100 | 52.36 | 252 | 46.49 | 68 | 46.9 | 22 | 46.81 | 60 | 56.6 | 91 | 47.64 | 290 | 53.51 | 77 | 53.1 | 25 | 53.19 | 0.599 |
| Able to control irritations | 42 | 47.73 | 93 | 49.47 | 228 | 45.78 | 90 | 48.65 | 36 | 49.32 | 46 | 52.27 | 95 | 50.53 | 270 | 54.22 | 95 | 51.35 | 37 | 50.68 | 0.899 |
| Felt they were on top of things | 63 | 39.87 | 124 | 54.87 | 232 | 46.49 | 55 | 53.4 | 13 | 32.5 | 95 | 60.13 | 102 | 45.13 | 267 | 53.51 | 48 | 46.6 | 27 | 67.5 | 0.008 |
| Angered by things out of their control | 53 | 46.09 | 92 | 54.44 | 220 | 42.72 | 86 | 51.5 | 38 | 56.72 | 62 | 53.91 | 77 | 45.56 | 295 | 57.28 | 81 | 48.5 | 29 | 43.28 | 0.022 |
| Could not overcome difficulties piling up | 68 | 47.22 | 102 | 49.51 | 216 | 44.63 | 73 | 49.32 | 30 | 58.82 | 76 | 52.78 | 104 | 50.49 | 268 | 55.37 | 75 | 50.68 | 21 | 41.18 | 0.314 |

Supplementary Table 3. Factors Associated with High Stress among Students Practicing Mindfulness and Therapeutic Yoga Practices (Sorig Zhiney and Luejong) program.

| Characteristics* | Total | Unadjusted analysis | | | Adjusted analysis | | |
| --- | --- | --- | --- | --- | --- | --- | --- |
|  |  | OR | 95% CI | P value | Adjusted OR | 95% CI | P value |
| Age (years) |  |  |  |  |  |  |  |
| ≥14 to <16 years | 66 | Ref |  |  |  |  |  |
| ≥16 to <18 years | 208 | 1.431 | 0.301 – 6.799 | 0.652 |  |  |  |
| ≥18 years | 216 | 2.744 | 0.616 – 12.213 | 0.185 |  |  |  |
| Sex |  |  |  |  |  |  |  |
| Male | 227 | Ref |  |  | Ref |  |  |
| Female | 263 | 5.662 | 1.9333 – 16.583 | 0.002 | 4.26 | 1.33 – 13.60 | 0.014 |
| Class |  |  |  |  |  |  |  |
| Class IX | 169 | Ref |  |  |  |  |  |
| Class X | 143 | 0.988 | 0.295 – 3.312 | 0.985 | 0.46 | 0.11 – 1.94 | 0.288 |
| Class XI | 69 | 0.815 | 0.160 – 4.145 | 0.806 | 1.15 | 0.21 – 6.34 | 0.871 |
| Class XII | 109 | 4.368 | 1.6374 – 11.652 | 0.003 | 3.00 | 0.94 – 9.62 | 0.064 |
| Living arrangement |  |  |  |  |  |  |  |
| Parents | 151 | Ref |  |  |  |  |  |
| Boarding | 312 | 0.698 | 0.305 – 1.594 | 0.394 |  |  |  |
| Relatives | 25 | 1.173 | 0.241 – 5.076 | 0.842 |  |  |  |
| Not specified | 2 | 13.500 | 0.784 – 232.282 | 0.073 |  |  |  |
| Feeling disconnected from family |  |  |  |  |  |  |  |
| Never | 201 | Ref |  |  | Ref |  |  |
| Very often | 11 | 7.510 | 1.960 – 28.776 | 0.003 | 5.82 | 1.06 – 32.07 | 0.043 |
| Often | 44 | 1.729 | 0.587 – 5.088 | 0.320 | 1.79 | 0.47 – 6.80 | 0.393 |
| Sometimes | 223 | 0.312 | 0.110 – 0.885 | 0.029 | 0.26 | 0.08 – 0.82 | 0.021 |
| Receives pocket money |  |  |  |  |  |  |  |
| No | 41 | Ref |  |  |  |  |  |
| Yes | 439 | - | - | - |  |  |  |
| Distraction or increase in stress with phone use |  |  |  |  |  |  |  |
| Never | 122 | Ref |  |  |  |  |  |
| Yes, often | 44 | 1.435 | 0.342 – 6.018 | 0.621 |  |  |  |
| Sometimes | 206 | 1.304 | 0.482 – 3.529 | 0.601 |  |  |  |
| Rarely | 116 | 0.840 | 0.249 – 2.835 | 0.780 |  |  |  |
| Increase in stress with use of phone |  |  |  |  |  |  |  |
| Never | 99 | Ref |  |  |  |  |  |
| Yes, often | 56 | 0.997 | 0.278 – 3.571 | 0.997 |  |  |  |
| Sometimes | 213 | 0.927 | 0.361 – 2.376 | 0.875 |  |  |  |
| Rarely | 119 | 0.334 | 0.084 – 1.330 | 0.120 |  |  |  |
| Good sleep |  |  |  |  |  |  |  |
| Yes | 400 | Ref |  |  |  |  |  |
| No | 90 | 1.561 | 0.641 – 3.798 | 0.326 |  |  |  |
| Appetite |  |  |  |  |  |  |  |
| Good appetite | 376 | Ref |  |  | Ref |  |  |
| Not having good appetite | 75 | 9.033 | 3.990 – 20.450 | <0.001 | 8.49 | 3.30 – 21.85 | **<0.001** |
| *Multivariable analysis was based on complete cases (n = 428) | | | | | | | |

Supplementary Table 4. Factors associated with high stress among students not practicing Mindfulness and Therapeutic Yoga Practices (Sorig Zhiney and Luejong) program.

| Characteristics* | Total | Unadjusted analysis | | | Adjusted analysis | | |
| --- | --- | --- | --- | --- | --- | --- | --- |
|  |  | OR | 95% CI | P value | Adjusted OR | 95% CI | P value |
| Age (years) |  |  |  |  |  |  |  |
| ≥14 to <16 years | 24 | Ref |  |  |  |  |  |
| ≥16 to <18 years | 192 | 1.12 | 0.13 – 9.31 | 0.913 |  |  |  |
| ≥18 years | 329 | 1.70 | 0.22 – 13.21 | 0.610 |  |  |  |
| Sex |  |  |  |  |  |  |  |
| Male | 251 | Ref |  |  | Ref |  |  |
| Female | 294 | 6.61 | 2.29 – 19.07 | 0.000 | 7.47 | 1.99 – 27.94 | 0.003 |
| Class |  |  |  |  |  |  |  |
| Class IX | 44 | Ref |  |  |  |  |  |
| Class X | 135 | 2.20 | 0.26 – 18.45 | 0.467 |  |  |  |
| Class XI | 135 | 0.61 | 0.05 – 6.90 | 0.689 |  |  |  |
| Class XII | 231 | 4.40 | 0.58 – 33.52 | 0.153 |  |  |  |
| Living arrangement |  |  |  |  |  |  |  |
| Parents | 160 | Ref |  |  |  |  |  |
| Boarding | 350 | 0.76 | 0.36 – 1.59 | 0.463 |  |  |  |
| Friends | 1 | - |  |  |  |  |  |
| Relatives | 28 | 0.48 | 0.06 – 3.88 | 0.494 |  |  |  |
| Not specified | 6 | - |  |  |  |  |  |
| Feeling disconnected from family |  |  |  |  |  |  |  |
| Never | 261 | Ref |  |  | Ref |  |  |
| Very often | 26 | 6.41 | 1.74 – 23.62 | 0.005 | 8.88 | 1.72 – 45.68 | 0.009 |
| Often | 34 | 7.56 | 2.37 – 24.08 | 0.001 | 3.71 | 0.68 – 20.16 | 0.128 |
| Sometimes | 218 | 2.94 | 1.19 – 7.29 | 0.020 | 3.41 | 1.01 – 11.51 | 0.048 |
| Receives pocket money |  |  |  |  |  |  |  |
| No | 29 | Ref |  |  | Ref |  |  |
| Yes | 510 | 0.26 | 0.09 – 0.75 | 0.013 | 0.26 | 0.05 – 1.38 | 0.114 |
| Distraction or increase in stress with phone use |  |  |  |  |  |  |  |
| Never | 166 | Ref |  |  |  |  |  |
| Yes, often | 97 | 2.04 | 0.79 – 5.21 | 0.138 |  |  |  |
| Sometimes | 161 | 0.94 | 0.35 – 2.49 | 0.897 |  |  |  |
| Rarely | 119 | 0.94 | 0.32 – 2.72 | 0.912 |  |  |  |
| Increase in stress with use of phone |  |  |  |  |  |  |  |
| Never | 161 | Ref |  |  |  |  |  |
| Yes, often | 82 | 2.29 | 0.85 – 6.19 | 0.101 |  |  |  |
| Sometimes | 185 | 0.99 | 0.37 — 2.64 | 0.987 |  |  |  |
| Rarely | 115 | 1.28 | 0.45 – 3.63 | 0.645 |  |  |  |
| Good sleep |  |  |  |  |  |  |  |
| Yes | 469 | Ref |  |  | Ref |  |  |
| No | 69 | 3.74 | 1.68 – 8.32 | 0.001 | 2.09 | 0.64 – 6.92 | 0.223 |
| Appetite |  |  |  |  |  |  |  |
| Good appetite | 414 | Ref |  |  | Ref |  |  |
| Not having good appetite | 98 | 6.85 | 3.03 – 15.48 | 0.000 | 4.11 | 1.42 – 11.90 | **0.009** |
| *Multivariable analysis was based on complete cases (n = 496) | | | | | | | |
